# Influenza Induced Cardiomyopathy: An Unusual Cause of Hypoxemia

**DOI:** 10.1155/2015/738146

**Published:** 2015-11-05

**Authors:** Abdullah Quddus, Maxwell Eyram Afari, Taro Minami

**Affiliations:** ^1^Department of Medicine, Memorial Hospital of Rhode Island, 111 Brewster Street, Pawtucket, RI 02860, USA; ^2^Alpert Medical School of Brown University, 222 Richmond Street, Providence, RI 02903, USA; ^3^Division of Pulmonary, Critical Care and Sleep Medicine, Memorial Hospital of Rhode Island, 111 Brewster Street, Pawtucket, RI 02860, USA

## Abstract

Influenza has considerable burden on public health funds. The complications of influenza can be devastating. We present a case of a 42-year-old woman with history of asthma who presented to the emergency room in winter with shortness of breath and general malaise and was found to be in hypoxemic respiratory failure. She was diagnosed with influenza and workup revealed severely depressed systolic cardiac function (ejection fraction of 25%). She was treated with oseltamivir and diuresis and regained cardiac function within a week. We review the pathophysiology and management of influenza induced cardiomyopathy.

## 1. Introduction

Influenza is known to cause serious illness, including severe hypoxemia and acute respiratory distress syndrome due to viral pneumonitis. We present a case of H1N1 influenza induced cardiomyopathy successfully treated with supportive treatment including diuresis.

## 2. Case Report

A 42-year-old woman with history of asthma presented to the emergency room in winter with complaints of worsening shortness of breath, general malaise, and diarrhea for a week. She denied fever or chills but endorsed sore throat. She was an exsmoker but denied alcohol or recreational drug usage. Physical examination revealed respiratory distress with increased work of breathing with oxygen saturation of 50% on ambient air. She was afebrile with blood pressure of 105/60 mmHg and a heart rate of 65 beats per minute. Chest auscultation was significant for diffuse crackles in both lung fields. Cardiovascular examination revealed normal heart sounds with no murmur, rubs, or gallops and no jugular vein distension. She did not have any peripheral edema.

**Figure 1:**
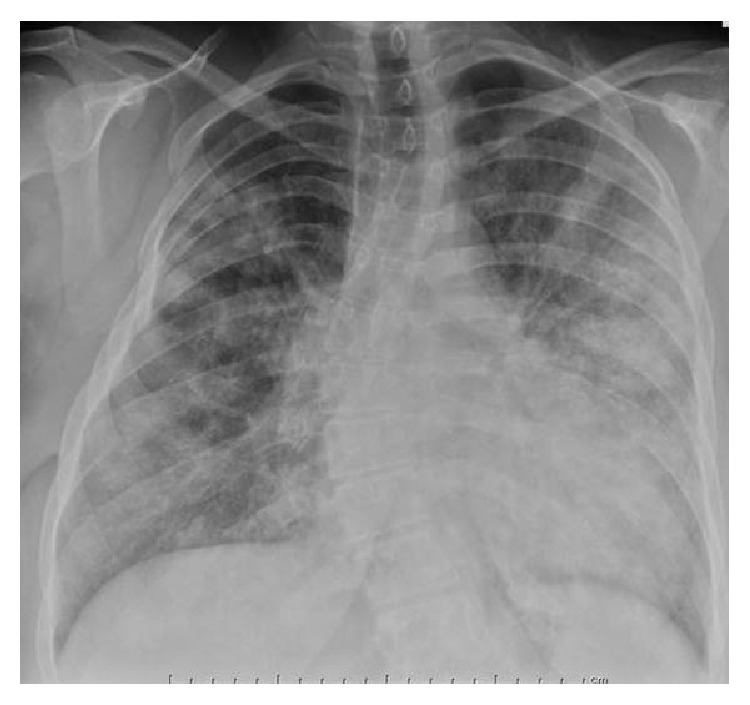
Showing the chest radiograph posteroanterior view. The cardiac silhouette is mildly enlarged but stable. The hilar structures are unremarkable. There are diffuse bilateral air space opacities. There is S-shaped thoracolumbar scoliosis.

**Figure 2:**
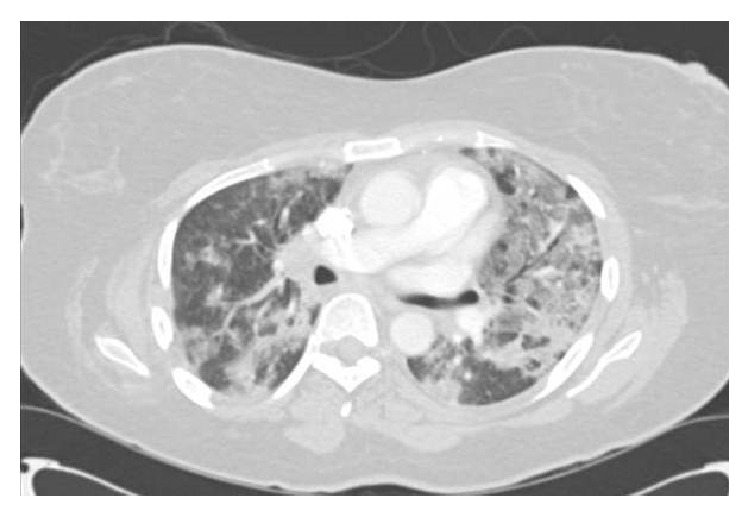
Showing computed tomography. There is moderate right hilar and mediastinal lymphadenopathy measuring up to 1.6 cm in short axis in the right hilum. The heart is moderately enlarged in size. There is a mosaic pattern of fairly dense opacity seen diffusely bilaterally.

Her laboratory data revealed a normal leukogram and hemogram. Rapid swab for influenza was negative. Basic natriuretic peptide was elevated at 626 pg/mL (reference < 100) and creatinine was 1.11 mg/dL (reference 0.42–1.06). The cardiac biomarker, troponin I peaked at 0.07 (reference 0–0.05 ng/mL). Electrocardiogram showed normal sinus rhythm with no ischemic changes. Chest radiograph showed diffuse bilateral patchy opacity, pulmonary vessel congestion, and mildly enlarged cardiac silhouette (Figure 1). Computed tomography of the chest showed bilateral ground glass opacities, more predominant in the left lobe (Figure 2).

She was admitted to the intensive care unit and was treated empirically with ceftriaxone (2 grams intravenously daily) and azithromycin (500 mg intravenously daily) for presumed community acquired pneumonia. Influenza was considered to be highly likely due to the season and epidemic and thus she was started on oseltamivir (75 mg twice daily). The urine test for legionella and pneumococcal antigen and blood cultures were negative.

Unfortunately, her respiratory status continued to deteriorate and required endotracheal intubation on day 5. Bronchoscopy with bronchoalveolar lavage did not yield any bacterial or fungal pathogens. An echocardiogram revealed a new onset cardiomyopathy with severely reduced systolic function (estimated ejection fraction of 25%) with severe global hypokinesis. She was started on intravenous diuresis with furosemide and angiotensin converting enzyme inhibitor (lisinopril). Polymerase chain reaction (PCR) of the nasopharyngeal swab was positive for influenza A. Further workup for HIV, coxsackievirus, adenovirus, thyroid function tests, and antinuclear antibodies and autoimmune and iron studies were unremarkable. A diagnosis of influenza related cardiomyopathy was made and the dose of oseltamivir was increased to 150 mg twice daily. She was tapered off all antibiotics.

The patient completed a 14-day course of oseltamivir. Her clinical condition gradually improved and she was successfully extubated on the eighth day of hospitalization. A repeat echocardiogram on day 8 of hospitalization revealed normal left ventricular function (ejection fraction 55%). Of note, she had never been vaccinated against seasonal flu.

## 3. Discussion

Influenza has considerable burden on public health funds with devastating complications. The highest risk can be found in immunosuppressed patients or those with chronic cardiopulmonary medical conditions, such as asthma. In the influenza pandemic of 1918-1919, healthy women between 20 and 40 years, as well as healthy men, had the highest morbidity and mortality [1, 2].

Known complication of influenza includes myopericarditis, rhabdomyolysis, and cardiogenic shock or death; however the most common complications are viral pneumonia and/or superimposed bacterial pneumonia. During the H1N1 pandemic in 2009, Martin et al. reported approximately 5% of affected patients having systolic dysfunction, of which more than 60% improved back to baseline [3]. This suggests that it is uncommon for systolic dysfunction to be seen and the natural history is for the recovery of the systolic function.

The pathophysiology of influenza induced cardiomyopathy is not completely understood. A cytokine storm is the key mechanism of vascular hyperpermeability in severe influenza [4]. Proinflammatory cytokines and endothelial cell dysfunction are suspected to contribute to the cardiac dysfunction. Pan et al. investigated the molecular mechanisms of influenza associated cardiac dysfunction and showed that inhibition of trypsin suppressed promatrix metalloproteinase and cytokine release which prevented ventricular dilation and thus improved cardiac function of mice infected with influenza virus A [5].

Despite the initial negative rapid flu swab, the clinical suspicion of influenza was still high. This qualitative testing, though very rapid (approximately less than 15 minutes), has low sensitivity. A meta-analysis of 159 studies during the H1N1 pandemic revealed a pooled sensitivity and specificity of 62.3% and 98.2%, respectively [6]. A subgroup analysis suggested that the sensitivity in adults (53.9%) is even lower than in children (66.6%). When there is high clinical suspicion, the rapid flu test cannot rule out influenza. Reverse transcriptase polymerase chain reaction is the most sensitive and specific testing modality of influenza [7].

In our patient the diagnosis of myocarditis was made based on the constellation of clinical symptoms and signs of heart failure, transient systolic dysfunction on echocardiogram, and the slight elevation of cardiac enzyme (some cardiac muscle injury). Based on the presentation, viral cardiomyopathy was on top of our differential diagnosis. Coxsackie, Epstein-Barr, Adenovirus, and HIV were ruled out. Isolation of the influenza virus from the myocardium is a big challenge; however positive influenza PCR highly suggested influenza as primary cause of the cardiomyopathy. Our broad diagnostic differential included autoimmune conditions like systemic lupus erythematosus, scleroderma, and Churg-Strauss which were promptly ruled out. Acute coronary syndrome, takotsubo cardiomyopathy, and metabolic (cocaine and hyperthyroidism) and noncardiogenic pulmonary edema (acute respiratory distress syndrome) were all considered but did not fit the clinical picture.

Rapid institution of antiviral agents like neuraminidase inhibitors (example: oseltamivir) has been shown to reduce complications [8]. Though the use of two times the standard dose (150 mg by mouth twice a day) is not validated this strategy has been previously described [9]. According to the Center for Disease Control and Prevention (CDC), some experts during the 2009 H1N1 pandemic recommended using double dose of oseltamivir for influenza patients who were severely ill and hospitalized.

## 4. Conclusion

HINI influenza can present as severe hypoxemia due to cardiogenic pulmonary edema, as opposed to ARDS. In cases of high clinical suspicion, a negative rapid test should not interfere with continued influenza treatment and/or workup. Vaccination is the primary strategy for the prevention and control of influenza. Vaccination in our patient could have prevented the illness and its complications and potentially ameliorated its severity. Asthmatic patients should receive appropriate vaccination, including flu shot. Prevention, in the end, is the goddess of good health, as Hygeia was the daughter of Asclepius.

## Supplementary Material

Echocardiogram demonstrates the cardiac Apical Four chamber view before and after treatment. The first 10 seconds of the video demonstrates severely reduced ejection fraction and global hypokinesis on admission. This is followed by 12 seconds video of the recovered ventricular function within a week of therapy.
